# Academic burnout profiles and their correlates: a latent profile analysis of Chinese university students

**DOI:** 10.3389/fpsyg.2026.1701455

**Published:** 2026-03-10

**Authors:** Yaojia Li, Yang Li, Xin Ye, Shizhen Yan

**Affiliations:** 1 School of Health, Fujian Medical University, Fuzhou, China; 2 Department of Educational Psychology, Faculty of Education, The Chinese University of Hong Kong, Shatin, Hong Kong SAR, China; 3 Faculty of Basic Science, Huizhou Health Sciences Polytechnic, Huizhou, China

**Keywords:** academic burnout, Chinese students, latent profile analysis, mindfulness, smartphone addiction

## Abstract

This study employed a person-centered approach to identify latent profiles of academic burnout among Chinese university students and to examine the associations between academic burnout profiles and smartphone addiction, sleep quality, and mindfulness. A sample of 2,948 Chinese university students was recruited to complete measures of academic burnout, smartphone addiction, sleep quality, and mindfulness. Latent profile analysis (LPA) was used to identify distinct burnout profiles, and multinomial logistic regression was used to analyze factors associated with profile membership. Three distinct profiles of academic burnout were identified: a Low Burnout profile (18.15%), a Medium Burnout profile (50.88%), and a High Burnout profile (30.97%). The profiles differed significantly on all correlates, with the high burnout group exhibiting the most severe smartphone addiction, the poorest sleep quality, and the lowest mindfulness. Regression analysis revealed that higher smartphone addiction and poorer sleep quality were significantly associated with membership in the Medium and High Burnout profiles relative to the Low Burnout profile, whereas higher mindfulness was significantly associated with lower likelihood of belonging to higher burnout profiles. Academic burnout among Chinese university students is a heterogeneous experience, with a majority falling into an at-risk or intermediate state. Smartphone addiction, poor sleep, and low mindfulness are associated with higher burnout risk. These findings highlight the need for universities to develop targeted, profile-based interventions to provide precise and effective mental health support. However, due to the cross-sectional design, causal relationships cannot be inferred.

## 1 Introduction

Academic burnout is a psychological syndrome characterized by emotional exhaustion, depersonalization, and a reduced sense of personal accomplishment, often stemming from chronic academic stress and a lack of motivation (Schaufeli et al., 2002). In China, intense educational competition, driven by rapid socioeconomic development and high demand for skilled professionals, has made students particularly vulnerable to this condition. While a significant body of research has explored burnout in high-pressure cohorts such as adolescents facing university entrance exams (Gao, 2023; Teuber et al., 2021) and medical students managing demanding coursework (Cheng et al., 2020; Xie et al., 2019), the broader university population remains comparatively understudied. As higher education becomes more widespread, burnout is emerging as a significant and pervasive challenge for university students across all disciplines.

Indeed, university students navigate a complex landscape of academic rivalry, career uncertainty, and social adaptation challenges, making them a high-risk group for academic burnout (Yu et al., 2020). Underscoring the scale of this issue, one large-scale study of 22,983 students found that approximately 55.16% experience some level of burnout, with nearly 5% reporting serious to very serious symptoms (Liu et al., 2023). The consequences are severe, as burnout not only compromises student mental health by increasing the risk of anxiety and depression (Liu X. et al., 2024; Liu W. et al., 2024), but also erodes learning motivation and academic engagement, ultimately contributing to poorer academic performance.

Among the factors associated with burnout, smartphone addiction has been identified as a critical correlate (Kates et al., 2018; Ye et al., 2023; Cheng and Wu, 2025). Defined as a behavioral addiction involving uncontrollable smartphone overuse that impairs psychosocial functioning (Liu et al., 2017), its link to burnout is well-documented. Studies suggest it may be related to burnout by fragmenting attention, reducing learning efficiency (Kaya, 2024), and diminishing intrinsic motivation (Zhang et al., 2021). More definitive evidence comes from recent longitudinal research confirming a bidirectional relationship, with smartphone addiction being a stronger predictor of subsequent burnout (Cheng and Wu, 2025).

Alongside behavioral risks, poor sleep quality is a significant correlate of academic burnout (Naderi et al., 2021; Zakiei et al., 2025). Mechanistically, sleep is essential for restoring the cognitive and emotional resources depleted during the day (Ben Simon et al., 2020). Chronic sleep deprivation has been linked to impaired functioning of the prefrontal cortex, a region critical for executive function and emotion regulation (van der Helm and Walker, 2009), which may impair emotion regulation, thereby exacerbating emotional exhaustion. This relationship may reflect a vicious cycle: burnout-induced anxiety could worsen insomnia, which in turn might deplete the psychological resources needed to cope with academic demands.

In contrast to these risks, mindfulness offers a key psychological resource for buffering against burnout. Defined as a relatively stable trait or dispositional capacity for non-judgmental, present-moment awareness (Brown and Ryan, 2003), mindfulness is consistently and negatively correlated with academic burnout (Cai et al., 2025; Liu X. et al., 2024; Liu W. et al., 2024). It is suggested to operate through two primary pathways: enhancing emotion regulation to potentially mitigate emotional exhaustion (Wheeler et al., 2017) and improving attentional control to help preserve a sense of accomplishment (Prakash, 2021; Sumantry and Stewart, 2021).

However, traditional research on these factors has predominantly adopted a “variable-centered” perspective, treating burnout as a single, continuous construct (Liu X. et al., 2024; Liu W. et al., 2024; Hu et al., 2024). This approach can oversimplify the complex reality of burnout by overlooking the heterogeneity within the student population. A “person-centered” approach, such as latent profile analysis (LPA), offers a more nuanced alternative by identifying distinct subgroups of students with different burnout profiles (Chirkowska-Smolak et al., 2023).

Adopting this person-centered perspective, the present study aims to investigate the heterogeneous nature of academic burnout. Its primary objectives are: (1) to identify the latent profiles of academic burnout among Chinese university students using LPA; (2) to compare differences in smartphone addiction, sleep quality, and mindfulness across these profiles; and (3) to examine the associations between these factors and profile membership. This study seeks to provide a scientific foundation for universities to develop more targeted and effective tiered intervention strategies.

## 2 Methods

### 2.1 Participants

This study utilized convenience sampling to recruit participants from universities in the Fujian and Guangdong provinces of China. Data were collected through an online survey platform. All participants were currently enrolled university students between the ages of 18 and 30. A total of 3,085 questionnaires were initially collected. To ensure data quality and validity, several exclusion criteria were applied: (1) responses from participants who failed embedded attention-check items (e.g., “For this item, please select A”) were excluded (*n* = 70); (2) incomplete questionnaires were removed (*n* = 35); and (3) outlier analysis was conducted on the total scores for the Academic Burnout Scale using a ±3 standard deviation (SD) criterion, resulting in the exclusion of 32 participants with extreme values. This process yielded a final sample of 2,948 valid responses, for an effective response rate of 95.56%. Among the 2,948 participants, 739 (25.07%) were men and 2,209 (74.93%) were women. The sample included students from all academic years: 1,277 first-year (43.32%), 722 second-year (24.49%), 709 third-year (24.05%), 190 fourth-year (6.45%), and 50 fifth-year or above (1.70%). This study received ethical approval from the institutional review board of Fujian Medical University (Approval No. FuYi Ethics Review [2024] No. 258).

### 2.2 Measures

#### 2.2.1 Smartphone addiction scale (SAS)

Smartphone addiction was assessed using the Chinese version of the SAS, originally developed by Kwon et al. (2013) and later revised for the Chinese context by Zhao et al. (2019). The 33-item scale measures six dimensions: withdrawal, overuse and tolerance, cyberspace-oriented relationship, importance, daily life interference, and emotional comfort. Items were rated on a 6-point Likert scale (1 = strongly disagree to 6 = strongly agree), with total scores ranging from 33 to 198. Higher scores indicate a greater risk or severity of smartphone addiction. The scale demonstrated excellent internal consistency in this study (Cronbach’s *α* = 0.92). Although the term “smartphone addiction” is used in this study to align with the original scale’s nomenclature, this construct is conceptually widely recognized as “problematic smartphone use” in the literature (Panova and Carbonell, 2018). It reflects maladaptive usage patterns characterized by daily life disturbance, withdrawal, and tolerance, rather than a clinically diagnosed dependence.

#### 2.2.2 Five facet mindfulness questionnaire (FFMQ)

Mindfulness was measured using the Chinese version of the FFMQ, originally developed by Baer et al. (2006) and revised by Deng et al. (2011). This 39-item scale assesses five facets of mindfulness: observing, describing, acting with awareness, non-judging of inner experience, and non-reactivity to inner experience. Items were rated on a 5-point Likert scale, and a total score was calculated by summing all item scores. Higher scores reflect a higher level of mindfulness. The scale has demonstrated strong reliability and validity; confirmatory factor analysis has shown ideal model fit, confirming its structural validity. The total score demonstrated good reliability in this study (Cronbach’s *α* = 0.90).

#### 2.2.3 Pittsburgh sleep quality index (PSQI)

Sleep quality was evaluated using the Chinese version of the PSQI, originally developed by Buysse et al. (1989) and adapted for the Chinese population by Liu et al. (1996). The 18 self-report items generate seven component scores: subjective sleep quality, sleep latency, sleep duration, habitual sleep efficiency, sleep disturbances, use of sleeping medication, and daytime dysfunction. Component scores are summed to produce a global score ranging from 0 to 21, where lower scores indicate better sleep quality. The scale showed good reliability in this study (Cronbach’s *α* = 0.823).

#### 2.2.4 Academic burnout scale (ABS)

Academic burnout was measured with the ABS, a widely used instrument for assessing burnout in Chinese university students developed by Lian et al. (2005). The 20-item scale comprises three dimensions: emotional exhaustion, improper behavior, and reduced personal accomplishment. Items were rated on a 5-point Likert scale (1 = totally disagree to 5 = totally agree). Crucially, items pertaining to personal accomplishment were reverse-coded, and the dimension was labeled “reduced personal accomplishment,” such that higher scores indicate lower accomplishment (i.e., higher burnout). Consequently, for all three dimensions, higher scores consistently represent higher levels of academic burnout. The scale demonstrated good internal consistency, with an overall Cronbach’s *α* of 0.87, and reliability coefficients for its subscales ranged from 0.70 to 0.81. To verify the structural validity of the scale in the current sample, a confirmatory factor analysis was conducted using Mplus 8.3. One item (Item 8) was removed due to a factor loading (0.22) below the acceptable threshold. To address the assumption of local independence, error correlations were allowed for items with high semantic similarity based on modification indices. The final measurement model demonstrated acceptable fit indices: *χ*^2^/df = 11.62, CFI = 0.89, TLI = 0.88, RMSEA = 0.07, and SRMR = 0.07. All factor loadings were significant (*p* < 0.001), confirming the three-factor structure.

### 2.3 Statistical analysis

LPA was conducted using Mplus 8.3 to identify distinct profiles of academic burnout. The three dimensions of the ABS (emotional exhaustion, improper behavior, and reduced personal accomplishment) served as indicators. To avoid local maxima and ensure solution stability, all models were estimated using 1,000 random sets of starting values and 200 final stage optimizations. A series of models (1–5 profiles) were estimated. Convergence was assessed by ensuring that the best log-likelihood value was replicated. The optimal number of profiles was determined based on: (1) lower values for the Akaike Information Criterion (AIC), Bayesian Information Criterion (BIC), and sample-size adjusted BIC (aBIC); (2) significant *p*-values (*p* < 0.05) for the Lo–Mendell–Rubin Likelihood Ratio Test (LMR-LRT) and the Bootstrap Likelihood Ratio Test (BLRT), indicating superior fit of a k-profile model over a k-1 profile model; (3) classification accuracy, evaluated using Entropy and Average Posterior Probabilities (AvePP), where AvePP > 0.70 indicates reliable classification (Nylund et al., 2007); and (4) theoretical interpretability and avoidance of extremely small classes (<5%).

To examine the associations between demographic (sex, academic year) and psychological variables (smartphone addiction, mindfulness, sleep quality) and profile membership while accounting for classification uncertainty, the R3STEP (three-step) procedure in Mplus was utilized. This method explicitly corrects for measurement error in class assignment within a multinomial logistic regression framework, providing unbiased parameter estimates (Asparouhov and Muthén, 2014; Vermunt, 2010). All effect estimates are reported as Odds Ratios (OR) with 95% Confidence Intervals (CI).

Descriptive statistics and univariate comparisons were performed using JASP 0.19.0.^1^ Prior to conducting parametric tests, the assumption of normality for continuous variables was evaluated. Given the large sample size of 2,948, normality was assessed using skewness and kurtosis values (Kim, 2013). All continuous variables exhibited skewness and kurtosis within acceptable ranges (absolute values < 2), justifying the use of these parametric methods. Chi-square tests were used to examine differences in demographic distributions (sex, academic year) across the identified profiles. One-way Analysis of Variance (ANOVA) was conducted to compare the profiles on continuous variables (burnout dimensions, sleep quality, smartphone addiction, and mindfulness).

## 3 Results

### 3.1 Descriptive statistics and preliminary group differences

A series of one-way ANOVAs were conducted to examine differences in the key variables across academic years. The results indicated a significant effect of academic year on academic burnout (*F*(4, 2,943) = 4.78, *p* < 0.001, $\eta_{p}^{2}$ = 0.01). Post-hoc tests revealed that first-year students reported significantly higher levels of academic burnout than third-year students (*p* < 0.001). A significant difference was also found for smartphone addiction (*F*(4, 2,943) = 4.44, *p* = 0.001, $\eta_{p}^{2}$ = 0.01), fourth-year students reported significantly higher levels of smartphone addiction than first-year students (*p* = 0.001). Furthermore, there was a significant main effect for mindfulness (*F*(4, 2,943) = 11.24, *p* < 0.001, $\eta_{p}^{2}$ = 0.02). First-year students had significantly lower mindfulness scores than both third-year (*p* < 0.001) and fourth-year students (*p* = 0.010). Lastly, a significant difference was observed for sleep quality (*F*(4, 2,943) = 6.43, *p* < 0.001, $\eta_{p}^{2}$ = 0.01). Post-hoc comparisons showed that third-year and fourth-year students reported significantly poorer sleep quality (i.e., higher PSQI scores) than first-year and second-year students (*p*s < 0.05). All descriptive statistics are presented in Table 1.

**Table 1:** Descriptive statistics and ANOVA results for key variables by academic year.

Variable	First-year	Second-year	Third-year	Fourth-year	Fifth-year	*F*	*p*
Academic burnout	54.70 ± 9.96	53.47 ± 9.82	52.79 ± 9.50	54.12 ± 10.08	54.56 ± 9.27	4.78	<0.001
Smartphone addiction	106.97 ± 26.07	109.25 ± 28.01	110.16 ± 27.68	114.88 ± 24.52	107.70 ± 25.28	4.44	0.001
Mindfulness	114.73 ± 9.26	115.71 ± 8.71	117.44 ± 8.72	117.02 ± 10.12	115.84 ± 7.39	11.24	<0.001
Sleep quality	4.84 ± 2.68	4.99 ± 2.84	5.38 ± 2.83	5.60 ± 2.53	4.96 ± 2.38	6.43	<0.001

Values are presented as M ± SD. For all *F*-tests, degrees of freedom were (4, 2,943). Lower scores on Sleep Quality indicate better sleep.

Independent samples *t*-tests were conducted to examine sex differences. The sample included 739 men (25.07%) and 2,209 women (74.93%). Women reported significantly higher academic burnout (54.21 ± 9.81) than men (52.98 ± 9.87, *t*(2,946) = 2.93, *p* = 0.003, Cohen’s *d* = 0.12). Similarly, women reported significantly higher levels of smartphone addiction (110.48 ± 26.03) than men (103.87 ± 28.83, *t*(2,946) = 5.81, *p* < 0.001, Cohen’s *d* = 0.25). Conversely, men reported significantly higher levels of mindfulness (116.06 ± 8.67) than women (115.69 ± 9.23, *t*(2,946) = 2.01, *p* = 0.045, Cohen’s *d* = 0.08). Finally, men reported significantly better sleep quality (i.e., lower PSQI scores) (4.64 ± 2.68) than women (5.20 ± 2.76, *t*(2,946) = −4.73, *p* < 0.001, Cohen’s *d* = −0.20).

### 3.2 Latent profile analysis of academic burnout

The fit indices for the LPA models are presented in Table 2. As the number of profiles increased, the AIC, BIC, and aBIC values progressively decreased. The LMR-LRT and BLRT results were significant (*p* < 0.001) for models ranging from two to four profiles, suggesting that adding profiles improved model fit.

**Table 2:** Fit indices of latent profile analysis on college students’ academic burnout.

Model	AIC	BIC	aBIC	Entropy	P_LMRT_	P_BLRT_	Class proportions (%)
Class 1	25,110.18	25,146.11	25,127.05	—	—	—	—
Class 2	23,583.96	23,643.85	23,612.07	0.70	<0.001	<0.001	39.32/60.68
Class 3	23,172.04	23,255.88	23,211.40	0.67	<0.001	<0.001	18.15/30.97/50.88
Class 4	22,771.24	22,879.04	22,821.85	0.75	<0.001	<0.001	16.83/1.19/31.04/50.95
Class 5	22,605.87	22,737.62	22,667.72	0.74	0.130	<0.001	8.26/10.34/1.15/34.84/45.40

AIC, Akaike Information Criterion; BIC, Bayesian Information Criterion; aBIC, Sample-Size Adjusted BIC; LMRT, Lo–Mendell–Rubin Likelihood Ratio Test; BLRT, Bootstrap Likelihood Ratio Test.

Although the four-profile solution yielded a higher entropy than the three-profile solution, it extracted a numerically small class accounting for only 1.19% of the sample. Conceptually, an inspection of the profile structure revealed that this additional class was merely a “level effect,” representing an extreme variant of the High Burnout group with a parallel shape, rather than a qualitatively distinct profile offering unique theoretical insight. Furthermore, such a small sample size could lead to instability in subsequent statistical analyses. Similarly, the five-profile model yielded a non-significant LMR value (*p* = 0.130) and an uninterpretable small class (1.15%).

Consequently, the three-profile model was selected as the optimal solution because it offered the best balance between statistical fit, model parsimony, and theoretical interpretability. While the entropy of the chosen three-profile model was 0.67, which is slightly below the conventional cutoff of 0.80, such values are not uncommon in large-sample research. To rigorously assess classification accuracy beyond entropy, we examined the AvePP. The AvePP for the three latent profiles were 0.869, 0.857 and 0.829, respectively. All values far exceeded the recommended threshold of 0.70 (Nylund et al., 2007), indicating that despite the modest entropy, the model assigned individuals to their respective profiles with high confidence.

**Figure 1:**
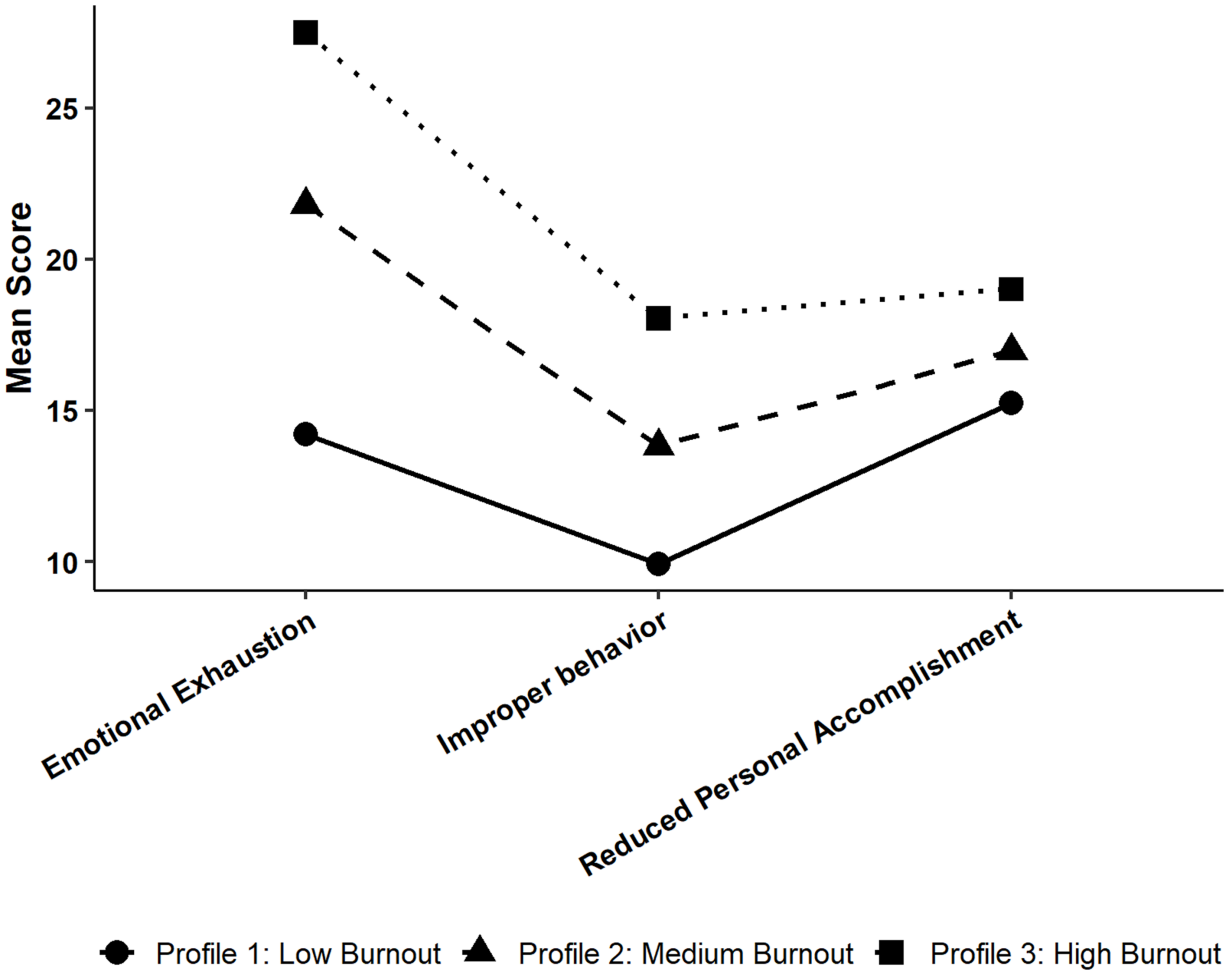
Standardized means of academic burnout dimensions across the three latent profiles. All indicators are coded such that higher scores represent higher levels of burnout.

To characterize the three latent profiles, a one-way ANOVA was conducted using profile membership as the independent variable and the three burnout dimensions as dependent variables. The results revealed significant differences among the three profiles on emotional exhaustion (*F*(2, 2,945) = 3,123.00, *p* < 0.001, $\eta_{p}^{2}$ = 0.68), improper behavior (*F*(2, 2,945) = 2,818.00, *p* < 0.001, $\eta_{p}^{2}$ = 0.66), and reduced personal accomplishment (*F*(2, 2,945) = 265.20, *p* < 0.001, $\eta_{p}^{2}$ = 0.15). The mean scores for each profile on these dimensions are illustrated in Figure 1.

The profiles were characterized as follows: Profile 1: Low Burnout (18.15% of the sample) was distinguished by the lowest scores on all three dimensions: emotional exhaustion, improper behavior, and reduced personal accomplishment. This group represents a psychologically well-adjusted and academically engaged cohort. These students exhibit stable moods and possess a very low risk of burnout. Profile 2: Medium Burnout (50.88% of the sample) was characterized by moderate scores across all dimensions. Notably, scores on emotional exhaustion were particularly pronounced relative to the other two dimensions within this group. This profile represents a large intermediate or at-risk population of students who are experiencing some degree of academic stress and emotional distress but have not yet reached a severe state. They are a key group for early attention and preventive intervention. Profile 3: High Burnout (30.97% of the sample) exhibited the highest scores on all three dimensions, indicating a comprehensive, high-risk burnout pattern. These students displayed severe emotional exhaustion, frequent improper behaviors, and an extremely low sense of academic accomplishment. This profile represents a group in a state of severe burnout, likely facing significant academic and psychological distress, and should be considered a high-priority population for psychological intervention and academic counseling.

### 3.3 Correlates of the latent burnout profiles

Demographic and psychological characteristics for each of the latent burnout profiles are presented in Table 3. A chi-square test revealed a significant difference in the distribution of academic years across the three profiles (*χ*^2^ = 29.19, *p* < 0.001). However, there was no significant difference in the distribution of sex across the profiles (*χ*^2^ = 1.35, *p* = 0.510).

**Table 3:** Demographic and psychological characteristics of the latent burnout profiles.

Characteristic	Low burnout	Medium burnout	High burnout	*χ*^2^/*F*	*p*
	*n* = 535 (18.15%)	*n* = 1,500 (50.88%)	*n* = 913 (30.97%)		
Demographics	*n* (%)	*n* (%)	*n* (%)		
Sex				1.35	0.510
Men	144 (19.49)	374 (50.61)	221 (29.91)		
Women	391 (17.70)	1,126 (50.57)	692 (31.33)		
Academic year				29.19	<0.001
First-year	212 (16.60)	618 (48.39)	447 (35.00)		
Second-year	132 (18.28)	382 (52.91)	208 (28.81)		
Third-year	158 (22.28)	368 (51.90)	183 (25.81)		
Fourth-year	27 (14.21)	101 (53.16)	62 (32.63)		
Fifth-year	6 (12.00)	31 (62.00)	13 (26.00)		
Psychological variables	M (SD)	M (SD)	M (SD)		
Smartphone addiction	85.37 (29.44)	108.26 (22.48)	123.47 (21.52)	439.70	<0.001
Mindfulness	122.40 (8.78)	116.30 (7.95)	111.10 (8.38)	322.70	<0.001
Sleep quality	3.75 (2.54)	4.93 (2.64)	6.03 (2.70)	130.00	<0.001

For categorical variables, values are presented as n (column %). The percentages for sex are calculated within each sex group. The percentages for academic year are calculated within each year group. Lower scores on Sleep Quality indicate better sleep.

A series of one-way ANOVAs were conducted to compare the three profiles on the key psychological variables. A significant difference was found for smartphone addiction (*F*(2, 2,945) = 439.70, *p* < 0.001, $\eta_{p}^{2}$ = 0.23). Post-hoc tests showed that the Low Burnout group (85.37 ± 29.44) scored significantly lower than the Medium Burnout group (108.26 ± 22.48), which in turn scored significantly lower than the High Burnout group (123.47 ± 21.52). Similarly, there was a significant difference in mindfulness levels (*F*(2, 2,945) = 322.70, *p* < 0.001, $\eta_{p}^{2}$ = 0.18). The Low Burnout group (122.40 ± 8.78) reported significantly higher mindfulness than the Medium Burnout group (116.30 ± 7.95), which was also significantly higher than the High Burnout group (111.10 ± 8.38). Finally, a significant difference was found for sleep quality (*F*(2, 2,945) = 130.00, *p* < 0.001, $\eta_{p}^{2}$ = 0.08). Post-hoc comparisons indicated that the Low Burnout group (3.75 ± 2.54) had significantly better sleep quality (i.e., lower PSQI scores) than the Medium Burnout group (4.93 ± 2.64), which in turn had significantly better sleep quality than the High Burnout group (6.03 ± 2.70).

### 3.4 Associations with burnout profile membership

To examine the associations between sex, academic year, sleep quality, smartphone addiction, and mindfulness and latent profile membership while accounting for classification uncertainty, the R3STEP procedure in Mplus was utilized. Continuous psychological variables were standardized (*Z*-scores) prior to analysis. The Low Burnout profile (Profile 1) served as the reference group. The results (see Table 4) indicated that sex was significantly associated with profile membership when comparing the High and Low Burnout profiles (OR = 0.60, *p* = 0.027), suggesting that female students had lower odds of belonging to the High Burnout group than males. Regarding academic year, compared to first-year students, both second-year (OR = 0.50, *p* = 0.007) and third-year (OR = 0.30, *p* < 0.001) students had significantly lower odds of being in the High Burnout group. However, regarding the Medium Burnout group, only third-year students showed marginally lower odds (OR = 0.68, *p* = 0.057).

**Table 4:** Multinomial logistic regression analysis of factors associated with membership in medium and high burnout profiles (Reference: Low Burnout).

Predictor	B	S.E.	OR	*p*	95% CI
Medium burnout vs. low burnout					
Sex (1 = Female)	−0.19	0.19	0.83	0.325	[0.57, 1.20]
Academic year (Ref: First-year)					
Second-year	−0.05	0.20	0.96	0.821	[0.64, 1.42]
Third-year	−0.38	0.20	0.68	0.057	[0.46, 1.01]
Fourth-year	0.41	0.43	1.51	0.333	[0.66, 3.47]
Fifth-year	0.51	0.90	1.67	0.570	[0.28, 9.82]
Psychological variables (*Z*-scores)					
Smartphone addiction	1.19	0.11	3.28***	<0.001	[2.67, 4.03]
Sleep quality (Poor)	0.35	0.10	1.42***	<0.001	[1.18, 1.72]
Mindfulness	−0.87	0.10	0.42***	<0.001	[0.34, 0.51]
High burnout vs. low burnout					
Sex (1 = Female)	−0.51	0.23	0.60*	0.027	[0.38, 0.94]
Academic year (Ref: First-year)					
Second-year	−0.69	0.25	0.50**	0.007	[0.31, 0.83]
Third-year	−1.20	0.25	0.30***	<0.001	[0.18, 0.49]
Fourth-year	−0.31	0.50	0.73	0.533	[0.28, 1.95]
Fifth-year	−0.27	0.98	0.77	0.785	[0.11, 5.22]
Psychological variables (*Z*-scores)					
Smartphone addiction	2.29	0.15	9.88***	<0.001	[7.33, 13.31]
Sleep quality (poor)	0.80	0.11	2.23***	<0.001	[1.78, 2.78]
Mindfulness	−1.84	0.14	0.16***	<0.001	[0.12, 0.21]

*N* = 2,948. Ref, Reference category; OR, Odds Ratio; CI, Confidence Interval. The Low Burnout profile served as the reference group. Sex was coded as 0 = Male, 1 = Female. Psychological variables (Smartphone Addiction, Sleep Quality, Mindfulness) were standardized (*Z*-scores) before analysis; thus, ORs represent the change in odds for a one-standard-deviation increase in the predictor. *p < 0.05, **p < 0.01, ***p < 0.001.

When comparing the Medium Burnout profile to the Low Burnout reference group, psychological variables emerged as significant correlates. Specifically, higher levels of smartphone addiction (OR = 3.28, *p* < 0.001) and poorer sleep quality (OR = 1.42, *p* < 0.001) were associated with increased odds of membership in the Medium Burnout group. Conversely, higher levels of mindfulness (OR = 0.42, *p* < 0.001) were associated with decreased odds.

Similar patterns were observed when comparing the High Burnout profile to the Low Burnout reference group, although the magnitude of the associations was notably larger. Higher levels of smartphone addiction (OR = 9.88, *p* < 0.001) and poorer sleep quality (OR = 2.23, *p* < 0.001) were robust correlates, strongly increasing the odds of membership in the High Burnout group. Mindfulness (OR = 0.16, *p* < 0.001) remained a key protective resource.

## 4 Discussion

This study employed a person-centered approach to identify distinct profiles of academic burnout among Chinese university students and to explore the associations between profile membership and key psychological variables. The results revealed three latent profiles—“Low Burnout,” “Medium Burnout,” and “High Burnout.” Across the Medium and High Burnout profiles, smartphone addiction and poor sleep consistently emerged as significant correlates, while mindfulness served as a key protective resource. These findings illuminate the heterogeneous nature of academic burnout and its associated psychological characteristics, offering important implications for the development of targeted intervention strategies.

### 4.1 The latent profiles of academic burnout

The average level of academic burnout reported in this study was consistent with findings from a large-scale study of 8,598 university students in China’s Anhui province (Ma et al., 2024), suggesting cross-regional similarity and stability in this phenomenon. Building on this, our latent profile analysis identified three distinct subgroups: a Low Burnout profile (18.15%), a Medium Burnout profile (50.88%), and a High Burnout profile (30.97%). This finding confirms the significant individual heterogeneity in how burnout is experienced and underscores the value of a person-centered approach.

The Low Burnout group represents a psychologically well-adjusted and academically engaged cohort. In contrast, the High Burnout group exhibited severe symptoms across all dimensions and constitutes a high-risk population requiring immediate support. Critically, the Medium Burnout group formed the majority of the sample. While not severely afflicted, these students already exhibit tendencies toward emotional distress and burnout, placing them in an at-risk state. This group is the core target for preventive efforts, as timely intervention may help mitigate the risk of symptom escalation. Compared to traditional methods that classify burnout based on total score cutoffs (Liu et al., 2023), the LPA approach provides a more empirically sound basis for accurately identifying student subgroups with different risk patterns, thus enabling more scientific screening and resource allocation in university settings.

### 4.2 Demographic correlates of burnout profiles

Regarding demographic characteristics, academic year emerged as a significant factor associated with profile membership, with first-year students being more likely to belong to the High Burnout profile than third-year students. This aligns with theories of student transition, which posit that first-year students face multifaceted challenges in adapting to a new environment, novel teaching methods, and different interpersonal dynamics, all of which can foster uncertainty and stress (Briggs et al., 2012). Third-year students, by contrast, have typically adapted to university life and possess more mature academic motivations and coping strategies.

In contrast to the descriptive statistics, which showed slightly higher mean burnout scores for women, the multivariate R3STEP analysis revealed a nuanced association regarding sex. Specifically, sex was a significant factor distinguishing the High Burnout profile from the Low Burnout group, with female students showing significantly lower odds of severe burnout compared to males (OR = 0.60). This suggests that while women may report higher average levels of general distress (as seen in the univariate analysis), men may be more vulnerable to the specific, severe symptom constellation characterizing the “High Burnout” profile when controlling for other psychological variables. While some studies report higher emotional exhaustion in women (Almalki et al., 2017; Romano et al., 2022), others have found men to be at higher risk for cynicism and depersonalization (Liu et al., 2023; Ma et al., 2024), which are core components of severe burnout. Our findings support the latter perspective, suggesting that male students may require targeted attention for severe burnout prevention, possibly due to differences in help-seeking behaviors or coping mechanisms that were not measured in this study.

### 4.3 Associations with burnout profile membership

The multinomial logistic regression results revealed that smartphone addiction and poor sleep quality were significant correlates of membership in both the Medium and High Burnout profiles, whereas mindfulness served as a robust protective resource. Notably, the association between smartphone addiction and the High Burnout profile was stronger than that with the medium profile, suggesting a dose–response relationship. These findings can be integrated into the Job Demands–Resources (JD-R) framework (Demerouti et al., 2001) and the Interaction of Person-Affect-Cognition-Execution (I-PACE) model (Brand et al., 2016) to elucidate the underlying mechanisms.

Within the JD-R framework, smartphone addiction and poor sleep quality can be conceptualized as chronic academic “demands” that tax students’ psychological and physiological resources. Excessive smartphone use consumes limited attentional capacity, increases cognitive load, and disrupts study routines, thereby potentially accelerating resource depletion and increasing vulnerability to burnout (Chen et al., 2023; Li et al., 2024; Zhang et al., 2021). Poor sleep quality further reflects impaired recovery, reducing students’ ability to restore energy and cope with daily academic stressors (Almalki et al., 2017).

The specific role of smartphone addiction can be further understood through self-regulation models of behavioral addiction, such as the I-PACE framework (Brand et al., 2016). According to this model, excessive smartphone use is associated with deficits in inhibitory control and emotion regulation. These self-regulatory impairments may contribute to burnout by maintaining maladaptive usage patterns and undermining recovery processes (Brand et al., 2019), thereby reinforcing a cycle of escalating demands and diminishing resources.

In contrast, trait mindfulness functioned as a key personal resource. Unlike state mindfulness induced by short-term interventions, the trait mindfulness measured in this study reflects relatively stable individual differences in attentional regulation, non-reactivity, and non-judgment (Baer et al., 2006). Our findings indicate that students reporting higher levels of trait mindfulness were less likely to belong to the Medium and High Burnout profiles. Mechanistically, trait mindfulness may help individuals sustain focused attention on academic tasks (attentional regulation), reduce automatic negative emotional responses to stressors (non-reactivity), and mitigate harsh self-criticism when facing setbacks (non-judgment). This is theoretically consistent with the motivational process of the JD-R model, suggesting that personal resources may facilitate more adaptive coping with chronic academic demands, thereby helping to mitigate the accumulation of emotional exhaustion over time (Cai et al., 2025; Ye et al., 2023).

Furthermore, trait mindfulness may not only act as a generalized personal resource but could also exert a potential buffering function between academic stress and burnout. For instance, stable individual differences in mindfulness may attenuate the impact of academic demands on emotional exhaustion by reducing maladaptive rumination and enhancing regulatory flexibility (Gu et al., 2015; Roemer et al., 2015). Longitudinal studies are needed to further clarify whether trait mindfulness moderates the relationship between academic demands and burnout trajectories.

### 4.4 Implications for practice

By adopting a person-centered perspective, this study validates the heterogeneous structure of academic burnout and deepens the understanding of how factors like smartphone use, mindfulness, and sleep correlate with this complex construct. These findings provide a clear framework for universities to implement precise interventions. First, universities can use burnout scales for regular screening to identify students in the High and Medium Burnout profiles and establish a tiered early-warning system. Second, differentiated interventions should be implemented: high burnout students require high-intensity support, such as individual counseling, academic tutoring, and targeted interventions for sleep problems. For the large Medium Burnout group, preventative group workshops focused on stress management, emotion regulation, time management, and healthy lifestyles would be more appropriate. Finally, multi-dimensional strategies should be integrated campus-wide. This includes promoting “digital wellness” education, such as psychoeducation on notification management, app-usage tracking, and establishing study-time phone-free blocks, as well as increasing awareness of sleep hygiene. With regard to mindfulness, a tiered intervention approach is recommended: institutions should distinguish between short, embedded mindfulness exercises within the daily curriculum for the general student body, and structured, 8-week mindfulness-based interventions, such as mindfulness-based stress reduction, or specialized web-based apps tailored for high-risk students. Given the vulnerability of new students, special focus should be placed on supporting first-year students during their critical transition period.

### 4.5 Limitations

This study has several limitations. First, its cross-sectional design precludes causal inferences. Future longitudinal research should track the developmental trajectories of burnout profiles and their correlates over time. Second, the generalizability of the findings is limited by the reliance on convenience sampling from only two provinces of China and the gender imbalance (approximately 75% female). These characteristics may restrict the applicability of our results to male students or those in other regions of China. Future studies should prioritize more balanced and nationally representative samples. Third, all data were collected via self-report, which may be subject to social desirability bias and common method variance. Future studies could incorporate objective measures, such as tracking smartphone usage with an app or monitoring sleep with wearable devices. Fourth, while we controlled for basic demographics (sex, year) and key psychological variables, other potential confounders were not measured in this study, such as students’ major (e.g., medical vs. non-medical), academic workload, socioeconomic status, or living arrangements. These unmeasured factors could influence academic burnout and should be considered in future investigations. Finally, the LPA model’s Entropy value of 0.67, while acceptable, suggests that the boundaries between the profiles are not perfectly distinct. Despite the adequate classification accuracy (AvePP > 0.80) achieved in this study, future research could explore alternative model solutions or examine the possibility of profile transitions over time. A promising avenue for future work would be to develop and test the efficacy of an integrated intervention program (e.g., mindfulness training combined with sleep hygiene education) based on the findings of this study.

## 5 Conclusion

By employing LPA, this study demonstrates that academic burnout among Chinese university students is not a monolithic construct but is instead a heterogeneous experience, best characterized by three qualitatively distinct profiles: Low Burnout, Medium Burnout, and High Burnout. The research further confirms that a high level of smartphone addiction and poor sleep quality are critical correlates associated with increased vulnerability to severe burnout, whereas a high level of mindfulness serves as a key psychological resource for fostering resilience against it. These findings underscore the importance of adopting a person-centered perspective to understand academic burnout. They provide an empirical foundation for universities to shift from one-size-fits-all interventions to a model of precision support, where differentiated prevention and intervention strategies are tailored to the specific needs of students in different burnout profiles. Finally, it is important to note that due to the cross-sectional design, the direction of causality cannot be established. The associations found in this study suggest potential pathways that should be verified in future longitudinal research.

## Data Availability

The original contributions presented in the study are included in the article/supplementary material, further inquiries can be directed to the corresponding author.

## Footnotes

1
https://jasp-stats.org
